# Genome-wide SNP-based genomic diversity and population structure analysis in alpaca populations from Europe and Peru

**DOI:** 10.1093/g3journal/jkag157

**Published:** 2026-06-20

**Authors:** Kirsty Tan, Olusegun Olaniyi Adeniyi, Anna Letko, Eberhard Manz, Henrik Werner Wagner, Patrik Zanolari, Manuel More, Gustavo A Gutierrez-Reynoso, Cord Drögemüller, Gesine Lühken

**Affiliations:** Institute of Animal Breeding and Genetics, Justus Liebig University Giessen, Giessen 35390, Germany; Institute of Animal Breeding and Genetics, Justus Liebig University Giessen, Giessen 35390, Germany; Institute of Genetics, Vetsuisse Faculty, University of Bern, Bern 3012, Switzerland; Generatio GmbH, Heidelberg 69115, Germany; Animal Clinic for Reproduction and Neonatology, Justus Liebig University Giessen, Giessen 35392, Germany; Clinic for Ruminants, Vetsuisse Faculty, University of Bern, Bern 3012, Switzerland; Universidad Nacional Agraria La Molina, Facultad de Zootecnia, Lima 15024, Peru; Universidad Nacional Agraria La Molina, Facultad de Zootecnia, Lima 15024, Peru; Department of Animal Science, Iowa State University, Ames, IA 50011-1130, United States; Institute of Genetics, Vetsuisse Faculty, University of Bern, Bern 3012, Switzerland; Institute of Animal Breeding and Genetics, Justus Liebig University Giessen, Giessen 35390, Germany

**Keywords:** South American camelids, genetic diversity, population structure, runs of homozygosity, principal component analysis, coat color

## Abstract

This study aimed to analyze the genetic diversity and population structure of alpacas in Germany, Switzerland, and Austria (German-speaking regions, GSR) and to compare with that of the country of origin of the species (Peru). A total of 179 animals from GSR and 151 from Peru were genotyped with a species-specific 76k SNP array. The observed and expected heterozygosity was 0.305 and 0.311 for GSR and 0.310 and 0.312 for Peru. The mean *F*_ROH_ values were 0.029 for GSR and 0.023 for Peru. In general, results show that breeders in both analyzed regions efficiently maintain genetic diversity. Principal component analysis identified the GSR and Peru populations as separate from each other, but the relative proximity of both clusters indicates the shared genetic heritage. *F*_ST_ and XPEHH methods identified genomic regions under selection for traits such as coat color and adaptation. Genome-wide association studies comparing black and brown with white or gray alpacas identified associated genome regions containing the *ASIP* and *KIT* genes, respectively. The association of a recently identified keratin locus on chromosome 16 with differences in fleece type in alpacas was confirmed, while the putative causality of a *TRPV3* variant was rejected.

## Introduction

The largest population of alpacas (*Vicugna pacos*) is in Peru due to migration of their wild ancestor from North America through the Isthmus of Panama into South America about 3 million years ago (Hoffman 2003), with approximately 3.6 million (80% of the world alpaca population) located there today (Figueroa et al. 2023). The import of alpacas into Europe began around the 1970s. The European alpaca population size has increased over the past decade due to its importance in a variety of functions, particularly in fleece production. Alpaca fiber is popular due to its unique properties, and the specific fleece characteristics are used to distinguish between 2 fleece types, Huacaya and Suri (Jost et al. 2020). Due to the small number of imported animals that established the base population in Europe, these alpacas can be expected to have a significantly smaller gene pool, increasing their risk of inbreeding.

In terms of alpaca genetics and breeding, there are some similarities and differences between the German-speaking region (GSR) of Europe and the Peruvian alpaca populations of South America. The primary selection criteria for breeding animals in GSR and Peru are fleece yield and fiber quality (fineness, density, uniformity, and fleece weight). Selection of these breeding animals is done using objective measurements of fiber diameter, medullation, and staple length with additional consultation of herdbook and performance records (Neubert et al. 2021; Wurzinger and Gutiérrez 2022). Mating systems differ between the GSR and Peruvian alpaca populations. The use of artificial insemination is limited due to the anatomy of alpacas, so live cover is standard among the alpaca breeders (Jost et al. 2020). Extensive natural mating is common in Peru, but controlled mating is preferred by elite breeders. They also value selective matings to improve specific traits such as fiber quality or color (Wurzinger and Gutiérrez 2022). The main differences in the breeding system between the GSR and Peru are the scale of production and genetic resources. The Peruvian breeding system is large-scale and semi-extensive. In contrast, the GSR population is smaller and semi-intensive. Due to the limited number of imported alpacas, the current GSR population is assumed to be at a higher risk of inbreeding (Figueroa et al. 2023).

Huacaya alpacas are the dominant fleece type (90% of all alpacas), although preference for fleece type is based on their functionality. Huacaya fleece, which has a crimpy texture, is suitable for knitted textiles such as sweaters and scarves. Suri fleece, which has a silky and lustrous texture, is preferred for woven products like suits and shawls (Tan et al. 2024). Coat color preferences are related to the commercial breeding of alpacas. There is a strong preference for white fleece in the commercial sector due to the versatility of this color. However, due to increased awareness of the blue-eyed white (BEW) phenotype and the availability of genetic testing to improve prediction of coat colors in offspring, phenotypes such as BEW are being selected against in breeding programs (Jones et al. 2019; Tan et al. 2022). Consequently, these traits are economically and functionally important.

Previous studies have focused on the genetic diversity and population structure of alpacas and other South American camelids in Peru and other parts of the world. Additionally, most of these genetic diversity studies used microsatellite markers since a SNP array was not developed for this species at that point (Paredes et al. 2012). Since the development of the first species-specific SNP array with 76,000 markers (Calderon et al. 2021), independent studies have reported associations of several genomic regions with different fleece traits (More et al. 2023; Tan et al. 2024).

Alpacas are a highly adaptable species, having the ability to live and thrive in a variety of geographical landscapes (Figueroa et al. 2023). Their adaptation to high-altitude and low-oxygen environments has caused certain evolutionary alterations in their genome (Richardson et al. 2019). Recently, selection signatures analyses based on whole-genome sequencing data have highlighted several candidate genes such as *EPAS1*, *EGLN1*, *PPARA*, *NOTCH4*, and *RBPJ*, all of which are hypoxia-related genes that function in regulatory responses to low-oxygen environments (Richardson et al. 2019). Another study suggested candidate genes for adaptation to harsh environments, such as *BSN*, *RIC8B*, *ANTXR2*, *PRDM8*, and *FGF5*, and production traits such as *RIC8B* and *DAG1* (Pallotti et al. 2023).

The aim of this study was to analyze the genetic diversity and population structure of 2 alpaca populations from different regions of the world: alpacas bred in GSR (of Europe), and native alpacas from South America (Peru). SNP array genotyping data were analyzed to identify regions under selection that are of particular interest for production-related traits such as fleece type and color.

## Methods

### Sample and data collection

Samples from the GSR region were collected from farms with varying numbers of alpacas. The most common reasons for keeping these animals were for breeding, wool production, and as pets (Jost et al. 2020). In Germany and Switzerland, it was possible to select on average only 1 to 2 samples per farm to reduce the likelihood of related animals and to achieve the most representative GSR population. A total of 179 alpaca samples were collected from different farms in Germany (*n* = 59), Switzerland (*n* = 62), and Austria (*n* = 58) with approximately equal numbers of males and females. They represented a variety of coat color groups (Supplementary Table 1) and both fleece types (Huacaya and Suri). Coat color and fleece type of each animal were reported from the owner or noted by the person who collected the sample. Detailed individual pedigree records and photographs were available for some but not all animals. The material of the collected samples included blood, hair, and nasal swabs. After DNA extraction, all samples were genotyped with the Affymetrix 76k alpaca SNP array (NEOGEN, Ayr, Scotland). The markers were mapped to the VicPac3.1 reference genome (GCF_000164845.4) and annotation Release 102 (Calderon et al. 2021).

In addition to the 179 GSR alpacas, already existing Affymetrix 76k alpaca SNP array data from 151 alpacas from 6 farms with varying altitudes, climates, and landscapes in Peru (3 from the Pasco region and 3 from the Puno region) were obtained for comparison with the GSR samples. In the Pasco region, samples were from Gacocen (*n* = 21), Racco (*n* = 19), and SOGAMU (*n* = 28), respectively. In the Puno region, samples were from Pacomarca (*n* = 30), Mallkini (*n* = 30), and Quimsachata (*n* = 17). The Pacomarca farm is a scientific station with approximately 2,500 alpacas of different colors and fleece types that specializes in the genetic improvement of alpacas (Cruz et al. 2023). The Quimsachata farm is associated with the largest alpaca germplasm bank in the world, contributing to increased genetic diversity of alpacas (Peralta et al. 2025). All these Peruvian samples were from female Huacayas with white coat color.

Collection of blood samples in Switzerland was approved by the Cantonal Committee for Animal Experiments (Canton of Bern; permit BE71/19 and BE94/2022) and in Germany by the Veterinary Department of the Regional Council of Giessen (19 c 20 15 h 02 Gi 19/1 KTV 22/2020). The SNP genotyping data of Peruvian samples were obtained from a previous project (Accession S-BSST1186) and in accordance with the Peruvian National Law No. 30407, “Animal Protection and Welfare Law,” in effect in Peru since 2016 January 7. In Austria, only nasal swab samples were collected.

### Quality control analysis and description of datasets

After removal of unplaced markers (17,211), the remaining dataset contained 59,273 markers. We curated the initial dataset of 330 individuals on 59,273 markers to perform 2 sets of analyses (Supplementary Table 1). Dataset 1 was created for genetic diversity and population structure analysis of the GSR and Peru populations and included only Huacaya animals. Dataset 2 was used to perform genome-wide association studies (GWAS) on different traits of interest, and principal component analysis (PCA) of alpaca fleece types. The different analyses are summarized in Supplementary Table 2. The data were checked for relatedness within the GSR and Peruvian populations through identity-by-descent (IBD) analysis using the --*genome* function of PLINK v1.9 (Chang et al. 2015), and closely related animals were removed. Additionally, quality control (QC) pruning for Dataset 1 (marker call rate >95%, minor allele frequency >5%) was carried out using PLINK v1.9 (Chang et al. 2015). The resulting Dataset 1 included 258 animals (113 GSR, 145 Peru) and 49,099 chromosome-localized markers remaining for downstream analysis (Supplementary Table 3).

Dataset 2 included the Huacayas from both populations (124 GSR, 151 Peru) and the 39 Suri animals for GWAS on traits of interest such as different coat colors and fleece type (Huacaya vs Suri). After QC (marker call rate >90%, minor allele frequency <5%), Dataset 2 consisted of 314 animals with 57,866 markers (Supplementary Table 3).

### Population structure and genetic diversity analyses

Linkage disequilibrium (LD) pruning was performed on Dataset 1 using the PLINK v1.9 (Chang et al. 2015) *--indep-pairwise* function by excluding one of each pair of SNPs in high LD (*r*^2^ > 0.5) within a window size of 50 SNPs and with a window slider of 5 SNPs. Consequently, 36,070 markers remained for analysis of PCA, *N*_e_, admixture, *H*_o_, *H*_s_, and *F*_IS_.

PLINK v1.9 (Chang et al. 2015) was used for PCA comparing animals within and between the GSR and Peru populations in Dataset 1. Additionally, all animals in Dataset 2 were divided based on fleece type (Huacaya and Suri) regardless of the geographical population grouping for PCA regarding fleece type difference. All plots were generated using the *ggplot2* package (Wickham 2016) in the R environment v3.6.0 (R Core Team 2019).

Dataset 1 was used to perform the effective population size (*N*_e_) analysis; each population was analyzed separately using the SNeP v1.11 software (Barbato et al. 2015). The *N*_e_ trends from 13 generations ago were calculated for the GSR and Peruvian populations using the population size numbers of 113 for GSR and 145 for Peru, and the options *-alpha 2.2*, *-maf 0.05*, *-numBins 45*, and default *-recrate 1.0e008*. Ancestry for both GSR and Peru populations was inferred with ADMIXTURE (v1.3) under unsupervised models for *K* = 1 to 4, using cross-validation (CV) to select optimal *K* by the minimum CV error (Alexander et al. 2009). The ancestry proportion matrices were visualized in R using the pophelper package (Francis 2017). To better understand the population structure determined by LD, an LD decay plot was constructed in R with ggplot2 after LD determination of Dataset 1 using PLINK v1.9 options *-r2* to calculate pairwise *r*^2^ value, *--ld-window-kb* 1,000 to set maximum distance in kb for considering variant pairs, *--ld-window* 999,999 to set maximum distance in number of bases and ensure this does not override the --*ld-window-kb* settings, and --*ld-window-r2* 0 to ensure that all *r*^2^ values close to zero are reported. After binning distances between base pairs at 10,000 kb, the mid-point of distances and mean *r*^2^ were obtained for each bin and used for plotting LD decay.

For calculation of basic statistics including the observed heterozygosity (*H*_o_), expected heterozygosity calculated as subpopulation heterozygosity (*H*_s_), and inbreeding coefficient (*F*_IS_), the *basic.stats* function of the hierfstat package (Goudet 2005) was implemented in R. *F*_IS_ was calculated using the formula 1 − (*H*_o_/*H*_s_) for each population separately. To determine the 95% confidence intervals of *F*_IS_ values, we employed the *boot.ppfis* function of the hierfstat package.

Runs of homozygosity (ROH) were determined for both populations in Dataset 1. It was not pruned for LD; therefore, 49,099 markers remained for the ROH analysis, and the parameters were determined according to Meyermans et al. (2020) (Appendix 1). The determination for ROH analysis parameters was done based on established settings compiled from several different studies and adapted to the alpaca SNP array (Meyermans et al. 2020). ROH were determined for different lengths of run: 1 to 2 Mb, 2 to 4 Mb, 4 to 8 Mb, and >8 Mb. These 4 length classes were determined as the more accurate measurement after comparison with length classes of 1 to 5 Mb, >5 to 10 Mb, and >10 Mb as the 4 classes covered short (1 to 2 Mb), intermediate (>2 to 4 Mb, >4 to 8 Mb), and long (>8 Mb) ROH regions more accurately. The frequency of ROH for each length class was compared between both GSR and Peru populations.

The genomic inbreeding coefficient based on ROH (*F*_ROH_) was calculated for all individuals per population by dividing the total length of all ROHs in the individual's genome by the total length of the autosomal genome (with a value of 1,563,654.764 kb) (McQuillan et al. 2008). Boxplots were constructed in R to visualize the resulting *F*_ROH_ values for both populations using the ggplot2 package.

### Signatures of selection analysis

Signatures of selection in Dataset 1 were calculated using *F*_ST_ and cross-population extended haplotype-based homozygosity (XPEHH). *F*_ST_ analysis was determined using pairwise *F*_ST_ values of each SNP comparing GSR and Peru groups using the *--fst* function in PLINK v1.90 (Chang et al. 2015). The *F*_ST_ values were *Z*-transformed to normalize its distribution using the formula: ZF_ST_ = (*F*_ST_ − *µ*)/*σ*, where *µ* is the mean and *σ* is the standard deviation of *F*_ST_ values, respectively (Eydivandi et al. 2021). Then, ZF_ST_ values were averaged over 500 kb nonoverlapping windows. The window-averaged ZF_ST_ values with a minimum of 5 SNPs were plotted across the scaffolds, and the top 1% windows of the empirical distribution were defined as putative selection regions. XPEHH was then applied to check the overlapping regions of similarity with *F*_ST_ analysis. Pairwise comparison between GSR and Peru for XPEHH scores was computed with the R package rehh (Gautier and Vitalis 2012) after phasing the data using fastPHASE 1.4 (Scheet et al. 2006). Haplotypes were inferred using fastPHASE v1.4 with default settings (20 EM starts, 25 iterations per start), cross-validated estimation of haplotype cluster number, and posterior haplotype sampling (*H* = 50), with simultaneous imputation of missing genotypes. To define selection signals, standardized XPEHH scores were transformed into pXPEHH = −log[1 − 2|ΦXPEHH − 0.5|] in which Φ XPEHH represents the Gaussian cumulative distribution function determined by the R function “pnorm.” The top 1% of the pXPEHH distribution equivalent to |XPEHH| score of ±2.79 were considered selection signals. To visualize the detected signatures of selection, XPEHH values were plotted against their physical position on each scaffold. Annotated genes within these genomic regions under selection were studied using the NCBI Genome Data Viewer (Genome Data Viewer 2024).

### Genome-wide association studies

In Dataset 2, GWAS comparing different fleece colors (59 solid-colored black [*n* = 21] and brown [*n* = 38] animals with 179 white animals or 19 gray animals) and fleece types (39 Suri and 275 Huacaya animals) were performed with linear mixed model including an estimated kinship matrix from centered genotypes using GEMMA 0.98.5 (Zhou and Stephens 2012). Default *-lmm 4* and *-gk 1* parameters were implemented with SNP significance evaluated using the score test. Bonferroni correction was applied to account for multiple testing across all SNPs, and the genome-wide significance threshold was defined as the probability of 0.05 divided by the total number of analyzed independent SNPs.

### Targeted genotyping of candidate variants

The *KIT* variant (NC_132988.1(VicPac4):g.84276093C>T; NW_021964157.1(VicPac3.1):g.38170962G>A; XM_031688000.1:c.391G>A) (Jones et al. 2019), proposed to be the causal variant for the dominant inherited classic gray phenotype (OMIA:000209-30538) and associated with the blue-eyed white phenotype, was genotyped in a total of 117 German and Swiss alpacas (15 gray and 102 nongray). In addition, the *TRPV3* variant (NC_133002.1(VicPac4):g.6676863G>T; NW_021964189.1(VicPac3.1):g.4265902G>T; XM_031685167.1:c.1423G>T) (Pallotti et al. 2024), which was suggested as a candidate variant for the Suri fleece type (OMIA:002841-30538), was genotyped in 175 animals (127 Huacayas, 48 Suris). Both variants were genotyped with Sanger sequencing after amplification with AmpliTaqGold360Mastermix (Thermo Fisher Scientific), on an ABI3730 capillary sequencer (Thermo Fisher Scientific) using the previously reported primers for the *KIT* variant (Tan et al. 2022) and similarly for the *TRPV3* variant using the following primers: forward 5′-ACCCTTGCCTTCCTTACTCC-3′ and reverse 5′-GATGGAGGGACGGGTAGATG-3′. The observed and expected frequencies for the *TRPV3* locus were calculated using both the allele frequency and Hardy–Weinberg equilibrium (HWE) equations: p+q=1 and $p^{2}+2pq+q^{2}+=1$ (Mayo 2008), where *p* and *q* represent observed frequencies of G and T alleles, respectively. In addition, observed genotype frequencies were assessed for deviation from HWE with the “HardyWeinberg” package in R using the “HWChisq” function (Graffelman 2015).

## Results

### Genomic diversity and relatedness of native Peruvian and European alpacas

**Fig. 1.**
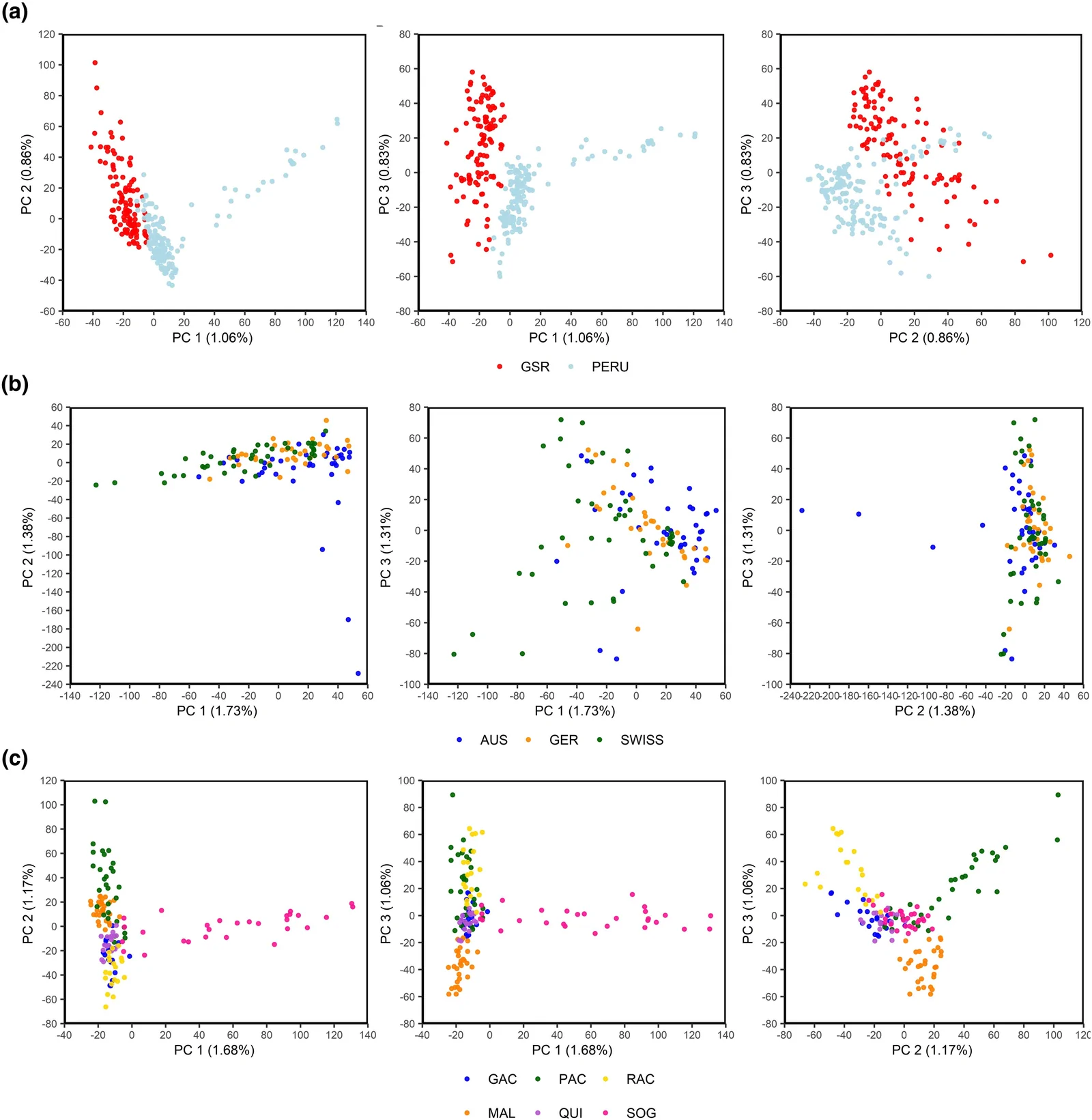
Principal component analysis (PCA) plot showing the distribution of a) GSR vs Peru populations based on the first 3 principal components. Individuals are colored by their region of origin (red, GSR; light blue, Peru); b) GSR populations based on the first 3 principal components. Individuals are colored by their region of origin (blue, Austria; orange, Germany; dark green, Switzerland); c) Peruvian populations based on the first 3 principal components. Individuals are colored by their region of origin (blue, Gacocen; dark green, Pacomarca; gold, Racco; orange, Mallkini; light purple, Quimsachata; pink, SOGAMU).

The PCA plot with the GSR (*n* = 113) and Peruvian (*n* = 145) populations showed separation by the first principal component only. However, clusters were not clearly distinct due to an overlap of individuals from the 2 groups (Fig. 1a). The population structure within the GSR population was analyzed using 38 Austrian, 33 German, and 42 Swiss animals (Fig. 1b). Most animals from the 3 countries were clustered closely together. Several outliers were seen among the Austrian and Swiss samples. Furthermore, PCA of the 6 Peruvian farms (Fig. 1c) showed animals from the SOGAMU farm mostly form a separate cluster within the first principal component. Additionally, the second and third principal components revealed a slight substructure of samples from the other farms.

The PCA of the Huacaya and Suri types using Dataset 2 was performed with an increased number of animals compared to the PCA in a previously published report (Tan et al. 2024). The repeated analysis reproduced the previous results of the 2 fleece types overlapping within the European population strengthening the hypothesis of 2 varieties in a single breed, rather than 2 separate breeds (Supplementary Fig. 1a), while the Peruvian cohort of only Huacaya animals formed a more distinct cluster as already shown in the Dataset 1 analyses.

**Fig. 2.**
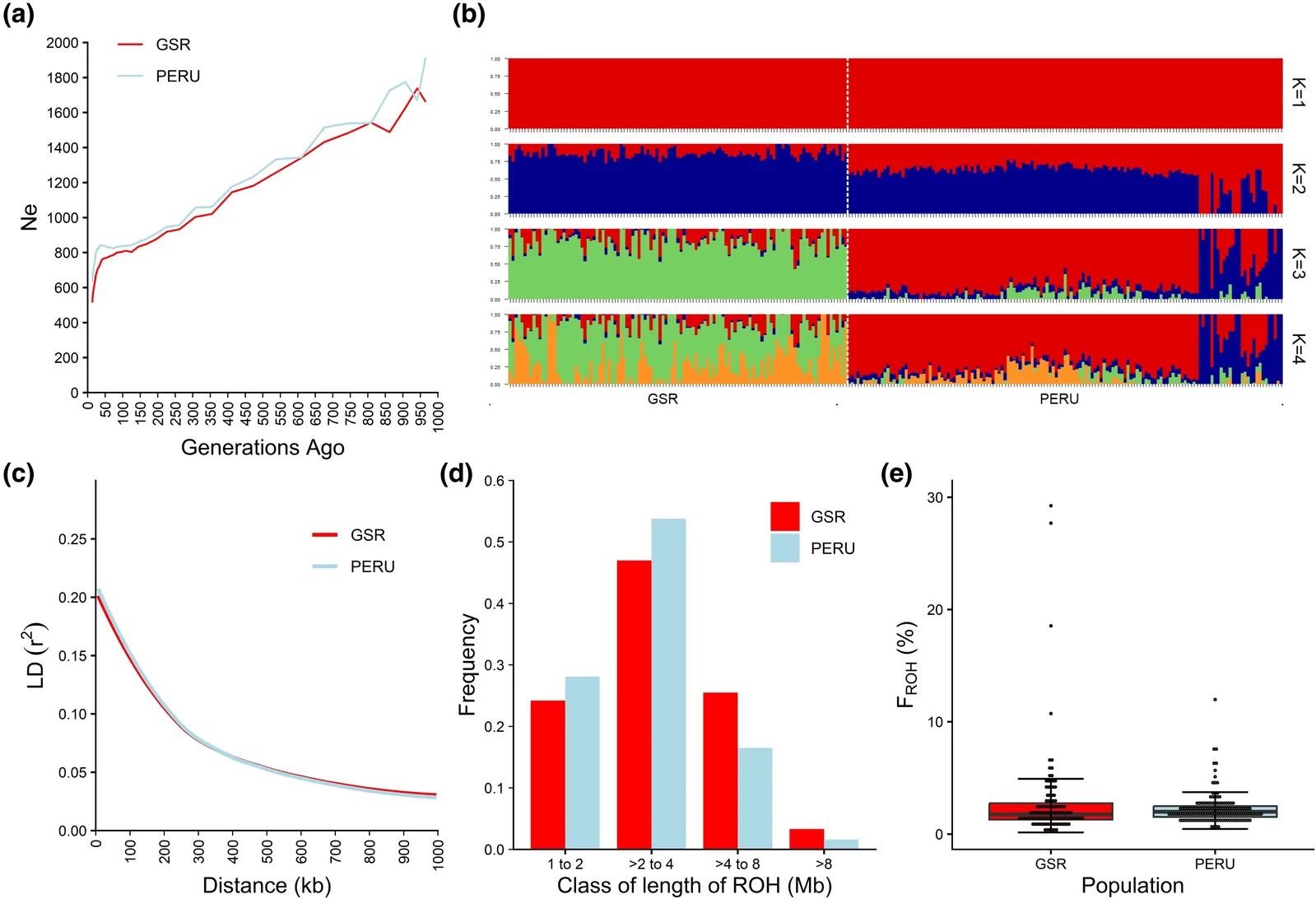
a) Trends of effective population size (*N*_e_) over generations for GSR vs Peru populations. b) Admixture plot for GSR vs Peru populations with an optimal *K* = 1. Colors represent ancestral clusters. Note that in the Peruvian cohort, 2 clusters are visible. c) The decay of LD (*r*^2^) at different genetic distances for GSR and Peru populations. d) Frequency distribution of the number of ROH in varying class lengths for GSR and Peru populations. e) Box plots of within-population distribution of runs of homozygosity-based inbreeding coefficient (*F*_ROH_) for GSR and Peru populations. Red represents GSR while light blue represents Peru for plots a, c, d, and e.

The effective population size (*N*_e_) trends starting at 13 generations ago were estimated and compared between the 2 populations (Fig. 2a). In general, *N*_e_ trends were higher in the Peruvian population. At 13 generations ago, the *N*_e_ was 514 for GSR and 634 for Peru while *N*_e_ at 50 generations ago was 768 and 838 for GSR and Peru populations, respectively. Admixture cross-validation error increased from 0.537 at *K* = 1 to 0.538, 0.540, and 0.542 at *K* = 2 to 4, with *K* = 1 representing the optimal cluster for interpretation of ancestry. However, the admixture plot revealed possible structure between both Alpaca populations at *K* = 2 as represented by variations in the proportions of the 2 ancestry clusters in the GSR and Peru populations (Fig. 2b). At *K* = 2 the proportions of cluster 1 (red) observed in the admixture plot were approximately ≤0.25 for GSR and >0.25 for Peru. Regarding LD decay, average *r*^2^ in LD decreased with increasing marker distance between pairwise SNPs with a sharper decline over the first 300 kb in both populations (Fig. 2c). Moreover, observed declining LD trends across marker distance were similar for both populations.

The average values for genetic diversity parameters in the investigated GSR and Peruvian alpaca populations are shown in Table 1. The values for both populations were very similar. Additionally, the *H*_o_ (GSR ≈ 0.305 and Peru ≈ 0.310) was lower than the *H*_s_ (GSR ≈ 0.311 and Peru ≈ 0.312) for both GSR and Peru populations, resulting in positive *F*_IS_ values (GSR ≈ 0.018 and Peru ≈ 0.005). Genetic diversity parameters for individual populations within GSR and Peru showed similar levels of *H*_o_ and *H*_s_ across populations (Table 1). All the Peruvian farms except one showed a higher *H*_o_ than *H*_s_, resulting in negative *F*_IS_ values.

**Table 1.** Average values for genetic diversity parameters in the German-speaking region and Peru alpaca populations.

Population	*H*_o_	*H*_s_	*F*_IS_
GSR	0.3052	0.3107	0.0177 (0.0162 to 0.0190)
Austria	0.3062	0.3110	0.0155 (0.0135 to 0.0172)
Germany	0.3050	0.3095	0.0145 (0.0117 to 0.0163)
Switzerland	0.3047	0.3086	0.0128 (0.0106 to 0.0146)
Peru	0.3100	0.3115	0.0045 (0.0035 to 0.0055)
Gacocen	0.3129	0.3113	−0.0052 (−0.0073 to −0.0028)
Mallkini	0.3090	0.3089	−0.0006 (−0.0025 to 0.0015)
Pacomarca	0.3123	0.3105	−0.0058 (−0.0075 to −0.0037)
Quimsachata	0.3133	0.3115	−0.0061 (−0.0088 to −0.0030)
Racco	0.3093	0.3103	0.0034 (0.0007 to 0.0054)
SOGAMU	0.3092	0.3049	−0.0139 (−0.0160 to −0.0115)

*H*_o_, observed heterozygosity; *H*_s_, expected heterozygosity; GSR, German-speaking regions.

Four different length classes of ROH were determined across populations (Table 2). The total number of ROH was 2,858 for GSR and 3,223 for Peru. The highest number of ROHs was in the >2 to 4 Mb class (1,343 for GSR, 1,733 for Peru), with an average of 8.33 ROHs per individual for GSR and 7.16 for Peruvian animals. The lowest number of ROHs was in the >8 Mb class (95 for GSR, 53 for Peru), with an average of 3.28 ROHs per individual for GSR and 1.83 for Peruvian animals. The ROH frequency distribution is shown in Fig. 2d. Mean *F*_ROH_ for GSR and Peru was 0.029 and 0.023, respectively (Fig. 2e). The range of *F*_ROH_ values (minimum to maximum) for GSR was 0.001 to 0.292 while the range of *F*_ROH_ values for Peru was 0.005 to 0.120. In comparison, the *F*_IS_ values were 0.018 for GSR and 0.005 for Peru.

**Table 2.** Runs of homozygosity for German-speaking regions and Peru populations.

Population	Class length (Mb)	ROH number (*n*)	Frequency
GSR	1 to 2	691	0.242
GSR	>2 to 4	1,343	0.470
GSR	>4 to 8	729	0.255
GSR	>8	95	0.033
Peru	1 to 2	905	0.281
Peru	>2 to 4	1,733	0.538
Peru	>4 to 8	532	0.165
Peru	>8	53	0.016

GSR, German-speaking regions; ROH, runs of homozygosity.

The class lengths are divided into 4 categories: 1 to 2 Mb, >2 to 4 Mb, >4 to 8 Mb, and >8 Mb.

### Identification of genes related to coat color, fleece type, and adaptation

**Fig. 3.**
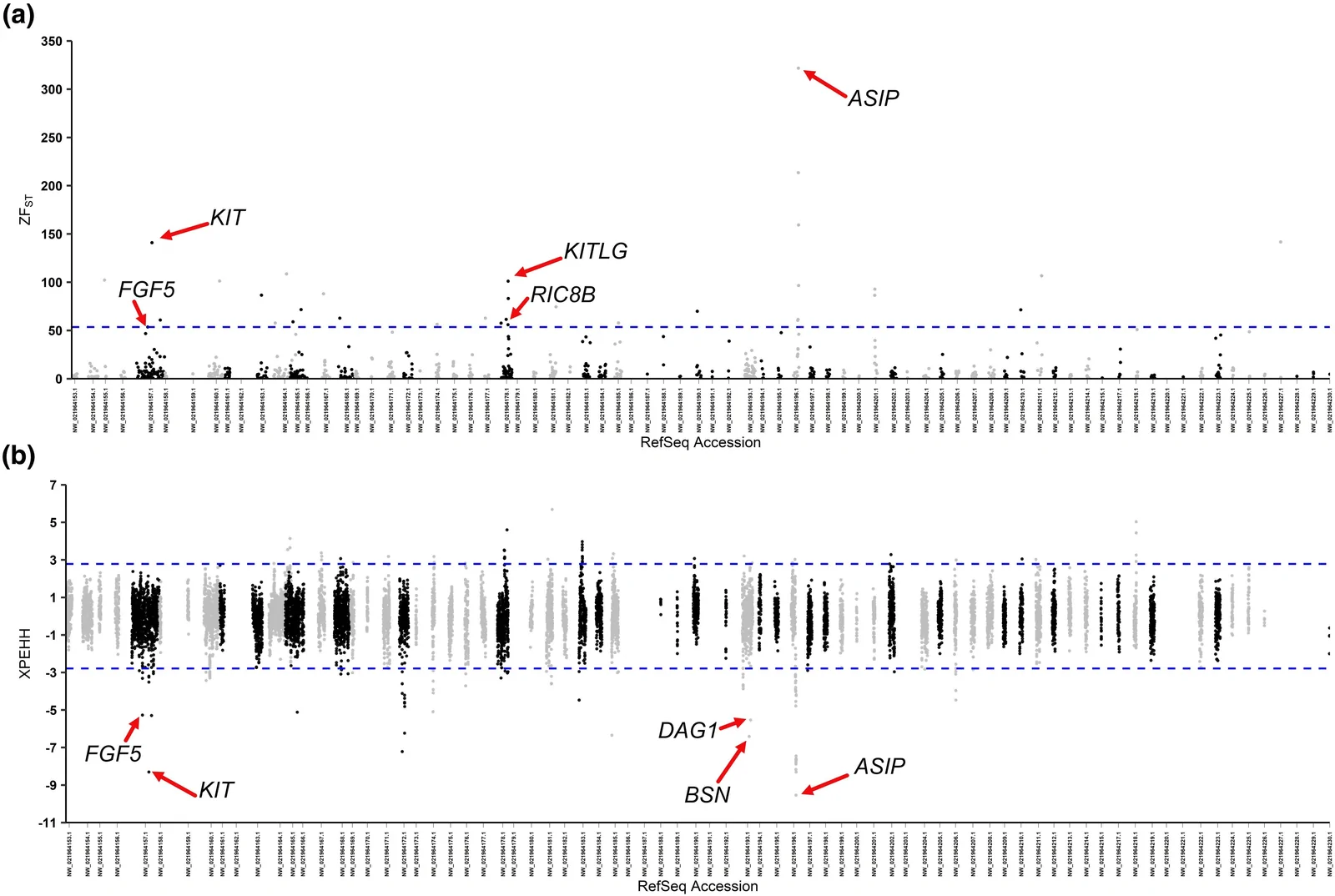
a) Manhattan plot of signatures of selection analysis (*F*_ST_). b) Manhattan plot of signatures of selection analysis (XPEHH). The *x* axes show the scaffolds in ascending order; all adjacent scaffolds of 1 color (gray or black) localize to the same chromosome. Selection signatures that harbor genes known to be related to coat color and adaptation traits are highlighted.

Signatures of selection analysis comparing the GSR and Peru populations revealed several regions of interest for coat color and fleece production traits. Using the *F*_ST_ method, the top associated marker was located on chromosome 19 within the NW_021964196.1 scaffold region of the *ASIP* gene (Fig. 3a) (Munyard 2011). XPEHH analysis (Fig. 3b) also indicated a positive selection for the Peruvian population, where the top associated marker was located in the same region of the NW_021964196.1 scaffold. An additional region that was putatively under selection pressure was noted from the *F*_ST_ analysis within the NW_021964178.1 scaffold (chromosome 12), where the top marker was located in the region of the *KITLG* gene (Shah et al. 2023). Both analyses identified a locus containing the *KIT* gene on chromosome 2 (scaffold NW_021964157.1). Besides this, further regions under selection with significant candidate genes for adaptation to harsh environments (Fan et al. 2020) were found on scaffold NW_021964157.1 of chromosome 2 (*FGF5*) for both *F*_ST_ and XPEHH, scaffold NW_021964178.1 of chromosome 12 (*RIC8B*) for *F*_ST_, and scaffold NW_021964193.1 of chromosome 17 (*BSN*) for XPEHH. A significant region under selection was also noted on scaffold NW_021964193.1 by XPEHH, containing the *DAG1* gene, that is previously reported to influence meat production traits (Pallotti et al. 2023).

**Fig. 4.**
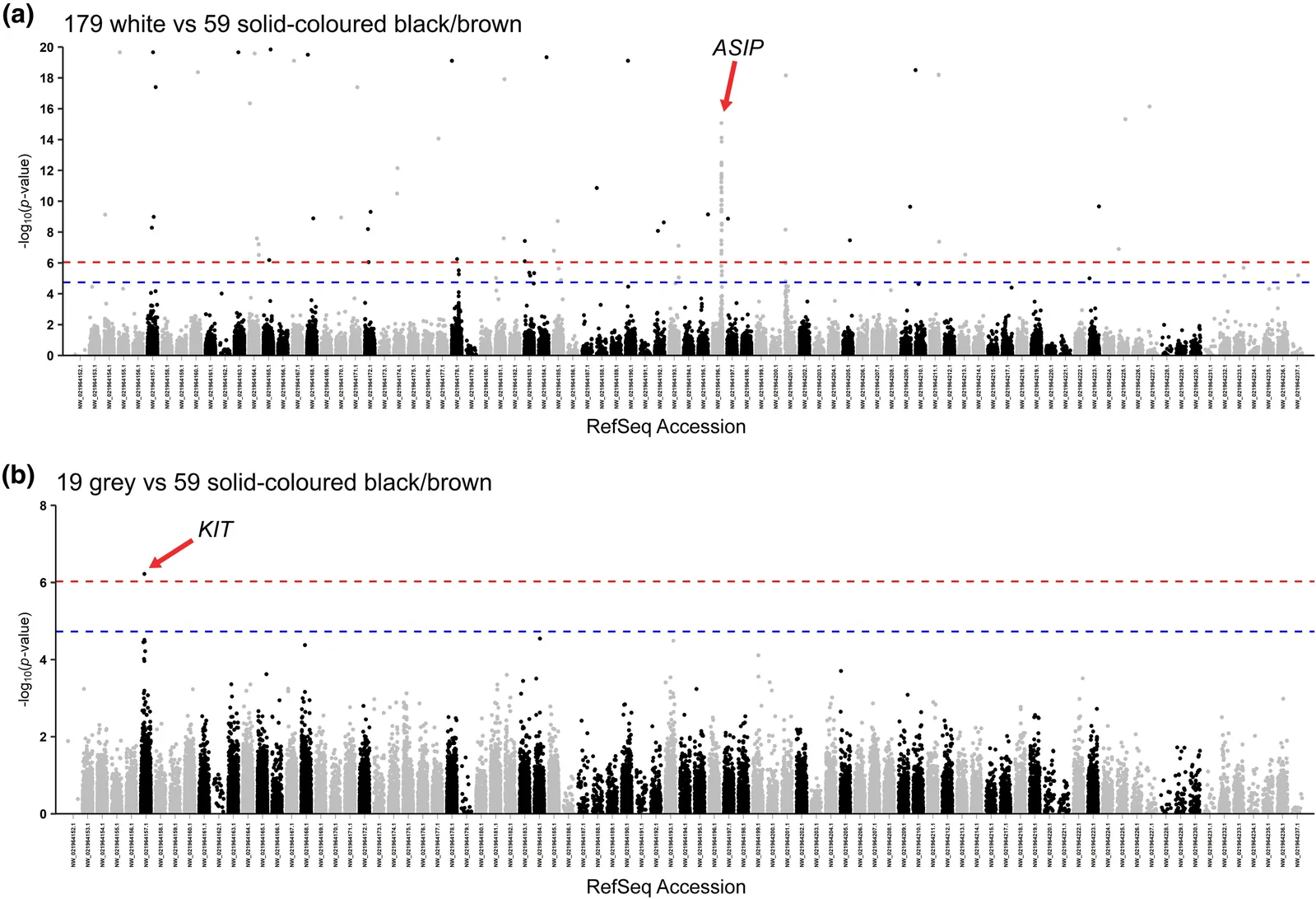
a) Manhattan plot of GWAS for white vs black and brown solid-colored animals highlighting the most associated markers at the *ASIP* locus. b) Manhattan plot of GWAS for gray vs black and brown solid-colored animals highlighting the most associated markers at the *KIT* locus. The red lines represent Bonferroni-corrected genome-wide significance thresholds (*P* = 9.0 × 10^−7^ and *P* = 9.4 × 10^−7^), while the blue lines correspond to the suggestive thresholds (*P* = 1.8 × 10^−5^ and *P* = 1.9 × 10^−5^). The *x* axes show the scaffolds in ascending order; all adjacent scaffolds of 1 color (gray or black) localize to the same chromosome.

GWAS analysis of 59 solid-colored black and brown vs 179 white animals further supported the selection signature finding as the most significant signal was located within the *ASIP* gene of scaffold NW_021964196.1 (Fig. 4a, Supplementary Table 4). A GWAS comparison of the same black and brown animals with 19 gray alpacas revealed a significantly associated locus on scaffold NW_021964157.1 containing the *KIT* gene (Fig. 4b, Supplementary Table 4).

The *KIT* variant was genotyped in 117 alpacas of various owner-reported coat colors. The genotyping revealed that most gray animals (14 out of 15) were heterozygous for the so-called classic gray variant. However, 15 nongray (white) animals were heterozygous. One gray animal had the homozygous reference genotype, the same as 87 nongray animals (Supplementary Table 5). The homozygous variant *KIT* genotype was absent.

Finally, the GWAS signal associating the recently identified (Tan et al. 2024) keratin locus with differences in fleece type was confirmed using an increased number of Suri and Huacaya animals (Supplementary Fig. 1b, Supplementary Table 4). The current GWAS based on extended case–control cohorts increased the power (*P* = 5.26 × 10^−11^ vs *P* = 3.14 × 10^−8^ before) of the previously reported significant association on chromosome 16 at scaffold NW_021964192.1 (Supplementary Fig. 1b) (Tan et al. 2024). The *TRPV3* variant was genotyped in 175 alpacas of both fleece types and was found with a genotype frequency of 0.77 for the homozygous reference genotype and 0.23 for the heterozygous genotype in Huacayas and 0.79 for the homozygous reference genotype and 0.21 for the heterozygous genotype in Suris, while no animal showed the homozygous variant genotype (Supplementary Table 6). There was no deviation from the Hardy–Weinberg equilibrium.

## Discussion

In this study, for the first time, 2 alpaca populations from different regions of the world, Europe and South America, were compared for genetic diversity and population structure based on genome-wide SNP data. Sampling aimed to reflect the diversity of the GSR and Peruvian populations. The PCA results showing clusters of GSR and Peru animals with a small degree of overlap reveal that these populations, while genetically distinct from each other, still share genetic heritage. Similarities in the ancestry of both populations are supported by the results of our admixture analysis which showed no clearly distinct subpopulation at optimal *K* (*K* = 1). The relative proximity of the 2 populations reflects the fact that alpacas were only brought into Europe approximately 50 years ago (Hoffman 2003). The outlier group in the Peruvian population (SOGAMU) might be more genetically distinct than the animals from the other Peruvian farms because of a founder effect from alpacas imported from Puno about 20 years ago, limited exchange of breeding alpacas due to different selection objectives of this farm such as selection for general appearance, conformation, fleece density, and fiber fineness, as personally reported by the owner. More et al. (2023) also found a separation of SOGAMU alpacas vs the others based on genomic relationships. The animals from the Pacomarca farm also might show more separation from the rest of the animals, although it is not reflected distinctly in the PCA plot, which could be because they are genetically selected for specific breeding goals (with a specific focus on reducing fiber fineness) in the Pacomarca Scientific Station. PCA of the fleece type cohorts further supports the theory of alpacas forming only a single breed with 2 fleece types, although the Peruvian all-Huacaya cohort forms a separate group; these differences are more likely based on geographical origin than fleece type. More Suri alpacas from Peru would be needed to further confirm this hypothesis.

For the GSR and Peru populations, with effective population sizes (*N*_e_: 13 generations ago) in the range of 500 to 650 are still considered genetically diverse as populations from an *N*_e_ of 50 and below are considered endangered (Mészáros et al. 2015). Regarding *N*_e_ values at 50 generations ago for both populations, we observed high values (768 and 838) indicating that both populations are genetically diverse with the Peruvian population showing higher *N*_e_ trends when compared to the GSR population (Fig. 2a). This indicates a higher diversity for the Peru population when compared to the GSR population and supports the lower *F*_IS_ for Peru reported in this study. We caution that these *N*_e_ values may be unreliable due to the inaccuracy in calculating both recent LD (<10 generations ago) and ancient LD (>4*N*_e_ generations in the past) as explained by Corbin et al. (2012). Furthermore, no difference in LD decay across distance was observed in both populations which explains the absence of a clearly defined structure between both populations as observed in both the PCA and admixture analyses.

The observed and expected heterozygosity was very similar for both populations, indicating a similar level of diversity within these populations. Individual diversity parameters showed the observed heterozygosity of 5 out of 6 Peruvian farms to be higher than their expected heterozygosity, showing that these populations have a low level of inbreeding and high genetic diversity as expected from the known breeding systems. The *F*_IS_ values of GSR populations were positive, reflecting a lower observed heterozygosity value than the expected heterozygosity value. A positive *F*_IS_ value usually indicates less genetic variation due to a higher degree of relatedness and potential inbreeding among individuals in a population. This often happens when the population size is smaller with restricted gene flow. Overall, diversity results indicate that breeders in both GSR and Peruvian regions effectively maintain genetic diversity and keep inbreeding low, aside from a few highly inbred animals. Previous microsatellite-based surveys of alpaca populations in South America have consistently detected high levels of genetic diversity and low to moderate population differentiation. A study of 2 Peruvian Huacaya populations found the expected heterozygosity of 0.77 (Figueroa et al. 2023). A similar high expected heterozygosity (0.73) was found in Bolivian alpacas along with weak genetic structure among domestic groups, as well as signature admixture with llamas (*F*_ST_ = 0.026) across productive systems (Echalar et al. 2020). In Peruvian Huacaya alpacas, a high allelic richness and heterozygosity for microsatellites were detected across breeding systems, with moderate population differentiation between managed and community herds. Principal coordinate and Bayesian clustering analyses indicated distinct genetic components among Suitucancha, Huayre, and the Quimsachata germplasm group, reflecting the influence of reproductive management on population structure (Peralta et al. 2025). In contrast, genome-wide SNP data analyzed in the current study provide higher resolution estimates of diversity and relatedness, allowing finer-scale detection of structure and differentiation, particularly among geographically or management-defined groups. These methodological differences imply that, while polymorphic microsatellites robustly capture broad patterns of genetic variation, biallelic SNP markers enable more nuanced characterization of genomic diversity and subtle population structure. Diversity parameters were measured in different breeds for other species such as cattle using data from SNP arrays, where the *H*_s_ ranged from 0.296 to 0.369 and *H*_o_ ranged from 0.303 to 0.375, with the lowest *H*_s_ and *H*_o_ values found in the least diverse cattle breed populations. These ranges show a high variability of expected and observed heterozygosity values among the different breeds (Schmidtmann et al. 2021). Compared to the range of values found in cattle due to a variety of different breeds, the values detected in more uniform species such as alpacas, are in the medium range with a smaller variation.

The length of the observed ROH segments may be used to distinguish between recent and earlier inbreeding and to draw conclusions about the population history (McQuillan et al. 2008; Keller et al. 2011). However, it is not possible to identify ROH of less than 1 Mb in length using low SNP array marker density (Purfield et al. 2012). Herein, both alpaca populations showed the lowest number of ROHs in the longest class, indicating a lower number of recent inbreeding events. The GSR alpaca population has a higher number of ROHs in the >4 to 8 Mb length class, which could be because of a smaller effective population size than found in the Peruvian population. These longer ROH segments reflect recent inbreeding, a plausible outcome due to the limited pool of unrelated mates. In contrast, ROH analysis comparing alpacas with other South American camelids using WGS data and shorter lengths revealed that alpacas have ROHs longer than >0.5 Mb more often than the llamas, guanacos, and vicunas, due to the fact that they have been inbred more recently than the other species (Pallotti et al. 2023).

Higher *F*_ROH_ values correspond to lower observed heterozygosity (*H*_o_) and are often positively correlated with the long-established Wright's inbreeding coefficient *F*_IS_, although *F*_ROH_ captures recent and ancient inbreeding more directly than pedigree or Hardy–Weinberg-based estimators. For the *F*_ROH_ outliers, pedigree data should be considered, and mates carefully chosen to avoid further inbreeding, although this requires detailed records or an established herd book with SNP data (Burren et al. 2016). In parallel, the relatively higher *F*_IS_ in the GSR compared to the Peruvian population, although both values are relatively low, points to mild but detectable nonrandom mating or population substructure. This is potentially driven by breeding practices such as the use of popular sires. The near-zero *F*_IS_ observed in Peru suggests mating patterns closer to Hardy–Weinberg expectations, consistent with larger population size and less intensive directional selection.

Genes under selection in both populations include regions where coat color genes such as *ASIP*, *KITLG*, and *MC1R* are present (Munyard 2011). All the Peruvian animals in this study were white, explaining the *ASIP* association in both signatures of selection and GWAS analyses. In alpacas the undesirable roan phenotype presents as depigmented fibers interspersed with the base coat color characterized by a mixture of white and colored fibers (Voß et al. 2022; Shah et al. 2023). Since coat color is an important trait in alpacas, influencing the market value of their fleece, variants in coat color genes indicate a selection driven by desirable economic traits. Negative XPEHH values observed in Peruvian alpacas indicate extended haplotype homozygosity consistent with long-term directional selection in the Andean environment. Among the strongest candidate genes, *FGF5* has been repeatedly associated with fiber length and fleece characteristics across mammals, including goats, sheep, and camelids, suggesting adaptive selection for enhanced thermal insulation under cold, high-altitude conditions (Fan et al. 2020). Other genes in selection signature regions indicate metabolic, neurological, and structural adaptations shaped by long-term selection in Peruvian alpacas. For example, *RIC8B* is linked to metabolic efficiency and environmental adaptation, *BSN* suggests a role for neurological and behavioral responses to stress, and *DAG1* is associated with muscle integrity and robustness in alpacas (Pallotti et al. 2023).

GWAS of gray vs solid-colored black and brown animals revealed a significant association with *KIT*, which plays a role in the classic gray phenotype in alpacas (Jones et al. 2019). Animals with available pedigree information that have gray animals within their ancestry should be tested for the *KIT* variant because of its link to the blue-eyed white phenotype (Tan et al. 2022) causing deafness in most animals with this phenotype as “cryptic gray” animals cannot be observed directly through their phenotype. In our study, 1 gray animal was homozygous for the reference allele, and 15 nongray (white) animals had the heterozygous *KIT* genotype, pointing to the challenges of correct phenotyping of animals as gray based on photos and limited pedigree data.

The improved GWAS for the Suri fleece type confirmed the significant association previously found on chromosome 16 (scaffold NW_021964192.1) locus containing a cluster of keratin genes. A recent study identified a variant in the *TRPV3* gene on a different scaffold (NW_021964189.1) of the same chromosome as a potential causal dominant-acting variant for the Suri fleece type (Pallotti et al. 2024). However, the genotypes of the *TRPV3* variant determined in 48 Suris and 127 Huacayas showed a very similar distribution in both groups (Supplementary Table 6). Therefore, this missense variant might potentially influence fiber structure and integrity but is most likely not the causal variant for the Suri fleece type.

Further whole-genome sequencing of alpacas, including long-read sequencing to identify possible structural variants, is required to determine the variants responsible for the observed differentiation in coat color and fleece type.

Despite the fact that the results presented in this study addressed the objectives of our investigation, we are aware of several limitations. First, the study relates to the utilization of an all-white, all-female, all-Huacaya cohort from Peru for comparison with a mixed-sex, mixed-color, mixed fleece-type population from GSR. In order to address this limitation, it would be advisable to undertake further analysis of the GSR population in order to rule out confounding factors. In addition, the modeling of fixed effects could be a viable option. Secondly, it was not possible to genotype the known candidate causal variants in all animals. It would have been preferable to consider these genotypes in the GWAS. Finally, enhancing the SNP marker density resolution, whether through sequence-level imputation or WGS-based approaches, would significantly augment the statistical power of genetic diversity and population structure analyses.

## Conclusions

This study improves our understanding of genome diversity and genomic relatedness of native Peruvian and nonnative European alpaca populations. It is the first study to compare population structure and genetic diversity of alpacas in Europe and Peru using SNP marker data. The results indicate high levels of genetic diversity for both populations, suggesting a low-risk status. Genetic diversity is crucial for the long-term sustainability of populations. In addition to pedigree information, breeders should use genomic data to more accurately maintain low inbreeding levels. Our previous finding that the Huacaya and Suri fleece types should not be considered as different breeds has been confirmed. The link between a recently identified keratin locus on chromosome 16 and fleece-type differences in alpacas was confirmed, whereas a causal role of the *TRPV3* variant was not supported.

## Supplementary Material

jkag157_Supplementary_Data

## Data Availability

SNP genotyping data are freely available at https://osf.io/b7jpt/. Supplemental material available at G3 online.
