# Supplementary material for: Genome-wide SNP-based genomic diversity and population structure analysis in alpaca populations from Europe and Peru
Source: G3 (Bethesda). 2026 Jun 20;16(9):jkag157. doi: 10.1093/g3journal/jkag157 (PMC13535496; doi:10.1093/g3journal/jkag157)
Supplement: Supplementary file 1 [file jkag157_supplementary_data.zip › Table_S2_G3-2026-406754.pdf]

Table S2. Summary of different analyses performed in each dataset

**Dataset 1**	**Dataset 2**
Heterozygosity (H_o, H_s)	Genome-wide association studies for coat colours and fleece type
Inbreeding coefficient (F_{IS})	Principal component analysis (Huacaya vs. Suri)
Runs of homozygosity (ROH)	
Genomic inbreeding coefficient (F_{ROH})	
Effective population size (N_e)	
Principal component analysis (GSR vs. Peru, GSR only, Peru only)	
Signatures of selection (F_{ST}, XPEHH)	
