# Supplementary material for: Genome-wide SNP-based genomic diversity and population structure analysis in alpaca populations from Europe and Peru
Source: G3 (Bethesda). 2026 Jun 20;16(9):jkag157. doi: 10.1093/g3journal/jkag157 (PMC13535496; doi:10.1093/g3journal/jkag157)
Supplement: Supplementary file 1 [file jkag157_supplementary_data.zip › Table_S3_G3-2026-406754.pdf]

Table S3. Details on quality control measures in datasets 1 and 2

Dataset	Populations	Initial samples (n)	Initial markers (n)	QC filters applied	Remaining samples (n)	Remaining markers (n)
1	GSR: Huacaya Peru: Huacaya	330	59,273	X-chromosome removal (1080); call rate >95%; MAF ≥5%; IBD PI_HAT <0.25	258	49,099
2	GSR: Huacaya, Suri Peru: Huacaya	330	59,273	Call rate >90%; MAF ≥5%; IBD PI_HAT <0.40	314	57,866

GSR = German-speaking regions, IBD = identity-by-descent, QC = quality control
