# Supplementary material for: Genome-wide SNP-based genomic diversity and population structure analysis in alpaca populations from Europe and Peru
Source: G3 (Bethesda). 2026 Jun 20;16(9):jkag157. doi: 10.1093/g3journal/jkag157 (PMC13535496; doi:10.1093/g3journal/jkag157)
Supplement: Supplementary file 1 [file jkag157_supplementary_data.zip › Table_S5_G3-2026-406754.pdf]

Table S5. Genotypes for *KIT* - classic grey variant (XP_006198200.1:c.376G>A) in 117 alpaca samples

Coat color	G/G	A/G	A/A
Grey	1	14	0
Non-grey	87	15	0
Total	88	29	0
