# Supplementary material for: Genome-wide SNP-based genomic diversity and population structure analysis in alpaca populations from Europe and Peru
Source: G3 (Bethesda). 2026 Jun 20;16(9):jkag157. doi: 10.1093/g3journal/jkag157 (PMC13535496; doi:10.1093/g3journal/jkag157)
Supplement: Supplementary file 1 [file jkag157_supplementary_data.zip › Table_S6_G3-2026-406754.pdf]

Table S6. Genotypes for *TRPV3* - putative fleece type associated variant (XM_031685167.1:c.1423G>T) in 175 alpaca samples

**Fleece type**	**G/G**	**G/T**	**T/T**
Huacaya	98	29	0
Suri	38	10	0
Total	136	39	0
